# Toxicity of Eight Insecticides on *Drosophila suzukii* and Its Pupal Parasitoid *Trichopria drosophilae*

**DOI:** 10.3390/insects15110910

**Published:** 2024-11-20

**Authors:** Huanhuan Gao, Yan Wang, Peng Chen, Ansheng Zhang, Xianhong Zhou, Qianying Zhuang

**Affiliations:** 1 Shandong Key Laboratory for Green Prevention and Control of Agricultural Pests, Jinan 250100, China; gaohuanhuan368@126.com (H.G.);; 2 Institute of Plant Protection, Shandong Academy of Agricultural Sciences, Jinan 250100, China; 3 Key Laboratory of Natural Enemies Insects, Ministry of Agriculture and Rural Affairs, Jinan 250100, China; 4 Rushan Agricultural and Rural Affairs Service Center, Rushan 264500, China

**Keywords:** spotted wing drosophila, sublethal effects, ecotoxicity, safety evaluation, integrated pest management

## Abstract

The pupal parasitoid *Trichopria drosophilae* (Hymenoptera: Diapriidae) has been evaluated as a biological agent of *Drosophila suzukii*. Integrated pest management strategies mostly rely on combined application of multiple insecticides and natural enemies. This study assessed the toxicity of eight common insecticides against *D. suzukii* in fruit orchards and the effects of semilethal and sublethal doses on *T. drosophilae*. The eight insecticides had higher toxicities to *D. suzukii* larvae with lower LC_50_ values than those for adults. Adults and larvae showed high susceptibility to emamectin benzoate, spinetoram, lambda-cyhalothrin, abamectin, and sophocarpidine. The median lethal doses (LC_50_) of lambda-cyhalothrin and imidacloprid to *T. drosophilae* adults were 60.41 mg/L and 100.58 mg/L, higher than the toxicities of the other six insecticides. Applying chlorantraniliprole, emamectin benzoate, sophocarpidine, abamectin, azadirachtin, and spinetoram resulted in low toxicity to *D. suzukii* pupae. However, the exposure of *D. suzukii* pupae or larvae to these insecticides at semilethal and sublethal doses decreased the parasitism or eclosion rate of *T. drosophilae*. These results improve our understanding of the effects of insecticide residues on *T. drosophilae* development and provide a basis for the combined use of chemical and biological options for managing *D. suzukii*.

## Simple Summary

*Drosophila suzukii* (Matsumura) (Diptera: Drosophilidae) is an important invasive pest of small soft-skinned fruits globally. The combined application of insecticides and natural enemies can effectively control *D. suzukii* and reduce chemical insecticide residues. The pupal parasitoid *Trichopria drosophilae* (Hymenoptera: Diapriidae) has been evaluated as a biological agent of *D. suzukii*. However, little is known about the toxicity of common insecticides in *T. drosophilae*. Thus, this study assessed the toxicity of eight common insecticides against *D. suzukii* in fruit orchards and the effects of semilethal and sublethal doses on *T. drosophilae*. Emamectin benzoate, spinetoram, lambda-cyhalothrin, abamectin, and sophocarpidine showed high toxicity in adults and larvae of *D. suzukii.* The toxicities of lambda-cyhalothrin and imidacloprid in *T. drosophilae* adults were higher than those of the other six insecticides. Exposure to chlorantraniliprole, emamectin benzoate, sophocarpidine, abamectin, azadirachtin, and spinetoram at semilethal and sublethal doses decreased the parasitism of *T. drosophilae* or eclosion of the next generation. In conclusion, some insecticides at the recommended dose applied for *D. suzukii* had no effect on the survival of *T. drosophilae* adults, but insecticide residues can affect *T. drosophilae* development.

## 1. Introduction

*Drosophila suzukii* (Matsumura) (Diptera: Drosophilidae) is an important invasive pest of small soft-skinned fruits globally [1]. More than 60 plant species have been identified as primary hosts, including strawberry, cherry, and blueberry [2,3]. *D. suzukii* is now well established throughout most subtropical, temperate, and boreal regions [4]. *D. suzukii* can cause 40–80% losses in fruit yield [5]. In recent years, it was estimated that *D. suzukii* caused USD 511 million in losses annually in western production regions of North America [6,7].

Although several preventive and control methods for *D. suzukii*, such as physical trapping, have recently been adopted [8], farmers still strongly rely on insecticide applications to protect fruit production. In previous studies, some common chemical insecticides in fruit orchards, such as beta-cypermethrin, pleocidin, emamectin benzoate, azadirachtin, and lambda-cyhalothrin showed higher toxicities in *D. suzukii* adults than imidacloprid, chlorantraniliprole, and avermectins [9,10]. In commercial orchards, various insecticides, including spinosad, cyantraniliprole, and lambda-cyhalothrin, are effective for the control of *D. suzukii* for at least 2 weeks until harvest, with very few damaged fruits [11]. Besides pesticide variation, larvae and adults of *D. suzukii* differ substantially in physiological characteristics and ecological habits and, therefore, are expected to exhibit differences in susceptibility and resistance to pesticides. For example, in cherry orchards in Shandong Province of China, the susceptibility to insecticides, such as pleocidin, emamectin benzoate, chlorantraniliprole, and lambda-cyhalothrin, is higher in *D. suzukii* larvae than in adults [12]. Spirotetramat can reduce the survival of adult flies but causes the death of all larvae under low-humidity conditions [13]. 

Long-term and repeated pesticide exposure increases the risk of resistance to pests and is a threat to human health and ecosystems [14,15]. In the fields in Shandong Province, the resistance of *D. suzukii* populations to five insecticides (imidacloprid, avermectins, pleocidin, emamectin benzoate, and lambda-cyhalothrin) did not increase, but the sensitivity to azadirachtin was decreased slightly [9]. Van Timmeren et al. [16] also compared the insecticide susceptibility of *D. suzukii* populations collected from insecticide-free blueberry sites in southwest Michigan, USA. After 3 years of monitoring, the resistance of *D. suzukii* to malathion and spinetoram increased slightly, but no change was registered in resistance to methomyl and zeta-cypermethrin. Therefore, the resistance of pests to insecticides depends on geographic population and the insecticide usage duration. It is necessary to monitor the susceptibility of *D. suzukii* to common insecticides regularly to identify safe, efficient control agents.

The effects of insecticides on natural enemies should also be considered when assessing insecticide safety. The parasitoids, such as *Trichopria drosophilae* (Hymenoptera: Diapriidae), *Pachycrepoideus vindemmiae* (Hymenoptera: Pteromalidae), *Trichopria anastrephae* (Hymenoptera: Diapriidae), *Ganaspis kimorum* (Hymenoptera: Figitidae), and *Leptopilina japonica* (Hymenoptera: Figitidae), have been evaluated as potential biocontrol agents of *D. suzukii* [17,18,19,20]. However, there is evidence for the toxicity of insecticides against these taxa. For example, Schlesener et al. [21] evaluated the toxicity of eight insecticides to *P. vindemmiae* and *T. anastrephae*, revealing higher sensitivity in *P. vindemmiae* than *T. anastrephae*. Malathion can cause 100% mortality in adult *T. anastrephae* [22]. Spinosyns (spinosad and spinetoram) and abamectin caused high *P. vindemmiae* mortality rates but did not affect *T. anastrephae* [20]. Moreover, sublethal or microdose insecticides may have long-term, trans-generational effects on enemies [23,24]. Passos et al. [25] showed that abamectin is highly toxic to nymphs of *Macrolophus basicornis* (Hemiptera: Miridae), a predator of *Tuta absoluta* (Lepidoptera: Gelechiidae). In contrast, methoxyfenozide, teflubenzuron, and chlorantraniliprole caused lower predator mortality and did not affect adult survival. Lisi et al. [26] reported that sublethal doses of cyazypyr and dimethoate negatively affect the success of parasitism and the fecundity of *T. drosophilae.* The pupal parasitoid *T. drosophilae* has recently been investigated for the biological control of *D. suzukii* in Europe [17]. In China, recent studies have focused on the artificial breeding of *T. drosophilae* and field application techniques in fruit orchards [27,28]. Accordingly, it is important to select insecticides that are highly effective against *D. suzukii*, without exhibiting detrimental side effects on *T. drosophilae*. 

Therefore, this study evaluated the toxicity of eight common insecticides toward *D. suzukii* at different developmental stages, as well as the acute toxicity to *T. drosophilae* adults and the effects on parasitism. The results provide a theoretical basis for the rational use of insecticides and for sustainable adoption of biological and chemical control against *D. suzukii* in China.

## 2. Materials and Methods

### 2.1. Insects

*Drosophila suzukii* pupae in cherry fruit were collected in June 2022 from a cherry orchard in Yantai (36.82° N, 120.69° E), Shandong Province, China. When the larvae developed into adults, one female and one male were fed by artificial diet and maintained in a climate-controlled growth chamber at 25 ± 0.5 °C, 60 ± 0.5% relative humidity, and a 16 h:8 h photoperiod for 4–5 generations. One liter of artificial diet was composed of 150 g mashed banana, 150 g mashed apple, 50 g corn flour, 50 g sucrose, 20 g yeast extract, 10 g agar powder, and water. Third-instar larvae, one-day pupae, and five-day-old adults were used for the following experiments.

*Trichopria drosophilae* emerged from parasitized *D. suzukii* pupae, which were collected in June 2022 from a grape orchard in Jinan (36.55° N, 116.79° E), Shandong Province, China. One pair of wasps and 20 *D. suzukii* pupae were placed in insect-rearing cages with mesh covers (20 cm × 20 cm × 20 cm) at 25 ± 0.5 °C, 60 ± 0.5% relative humidity, and a 16 h:8 h photoperiod. Additionally, 30% honey water was provided in the cage to supplement wasp nutrition. After 24 h of parasitism, the pupae were removed from the cage and new pupae were added for continued parasitism. Wasps that emerged after 17–20 days were the next generation. After 4–5 generations of parasitism, *T. drosophilae* adults were used for subsequent experiments.

### 2.2. Insecticides

Eight insecticides commonly used in fruit orchards in China were selected for this experiment, including six chemical insecticides (i.e., chlorantraniliprole (Diamides), spinetoram (Spinosyns), emamectin benzoate, abamectin (avermectins), lambda-cyhalothrin (Pyrethroids), and imidacloprid (Neonicotinoids)) and two botanical insecticides (i.e., sophocarpidine and azadirachtin). The concentrations of active ingredients, production details, and maximum recommended field dose are shown in Table 1.

### 2.3. Toxicity of Insecticides on Drosophila suzukii at Different Life Stages

For the larval toxicity experiment, the concentrations of eight insecticides (chlorantraniliprole, spinetoram, emamectin benzoate, lambda-cyhalothrin, imidacloprid, abamectin, sophocarpidine, and azadirachtin) are shown in Table 2. Eight insecticides were diluted to six concentrations progressively from the highest to the lowest concentration using distilled water. The solution volume of insecticides in each treatment was fixed to 100 mL. The solutions were discarded and placed into the toxic waste recycling bucket after treatment (insecticide solutions used in the following experiments were treated the same way). Next, in plastic Petri dishes (3.5 cm diameter) containing six concentrations of eight insecticides, 4 g of the artificial diet was soaked for 2 min. This time could ensure that 4 g of artificial diet was exposed evenly, according to the treatment time for larvae in the paper by Lisi et al. [26], modified in this study. Twenty second-instar larvae were placed on an air-dried insecticide-contaminated diet in each plastic Petri dish for 48 h. In order to avoid the effects of environmental factors on insect survival, all the larvae in this experiment were placed in a climate-controlled growth chamber at 25 ± 0.5 °C, 60 ± 0.5% relative humidity, and a 16 h:8 h photoperiod. Larvae treated with distilled water using the above method were designated as the control (0 mg/L). Each treatment had three replicates. The mortality of *D. suzukii* larvae was assessed by counting the dead larvae in each insecticide treatment.

For the pupal toxicity experiment, the concentrations of the eight insecticides are shown in Table 2. Eight insecticides were serially diluted to five concentrations by 25%, progressively from the highest concentration using distilled water. The volume of insecticides in each treatment was fixed to 100 mL. In order to know the insecticide concentration that the pupae can tolerate, we increased the maximum concentration, which was about equal to or higher than the recommended field dose. Twenty 1-day-old pupae were soaked for 2 min in each insecticide at five different concentrations. Pupae treated with distilled water using the above method were designated as the control (0 mg/L). All the pupae in this experiment were placed in a climate-controlled growth chamber in the above conditions. Each treatment had three replicates. The mortality of *D. suzukii* pupae was assessed according to the emerging adults in each insecticide treatment.

For the adult toxicity experiments, the concentrations of eight insecticides are shown in Table 2. The eight insecticides were serially diluted to six concentrations, progressively from the highest concentration using distilled water. The volume of insecticides in each treatment was fixed to 100 mL. Next, 4 g artificial diet was soaked for 2 min in rearing tubes (9.5 cm height and 1.5 cm diameter) with eight insecticides at six concentrations. Then, 20–30 adults (male/female = 1:1) were placed on an air-dried insecticide-contaminated diet in rearing tubes for 48 h. Adults treated with distilled water using the above method were designated as the control (0 mg/L). All the adults in this experiment were placed in a climate-controlled growth chamber in the above conditions. Each treatment had three replicates. The mortality of *D. suzukii* adults was assessed for each insecticide.

For each life stage, the concentration–mortality regression lines for all insecticides at the six above concentrations were determined on each stage (larvae, pupae, and adults). LC_10_ (10% lethal concentrations) and LC_50_ (50% lethal concentrations) values of eight insecticides, the confidence intervals (95%), and correlation coefficient (R^2^) were calculated according to the concentration–mortality regression lines. It should be explained that, in order to obtain a better regression curve, we conducted a pre-experiment with the same methods to select the maximum treatment concentration for *D. suzukii* larvae and adults. Almost all the tested larvae and adults died after treatment with the maximum concentration of insecticides. The raw data of the survival rate of larvae and adults exposed to eight insecticides at high concentrations are shown in Tables S1 and S2.

### 2.4. Acute Toxicity of Insecticides on Trichopria drosophilae

According to the maximum recommended field doses of eight insecticides were applied in orchards (Table 1), six concentration gradients were made using distilled water for each insecticide (Table 2). The highest concentrations of insecticides in this experiment were 10 times the maximum recommended field doses according to GB/T 31270.12-2014 (test guidelines on environmental safety assessment for chemical pesticides, Part 17: Trichogramma acute toxicity test) [29]. Additionally, 0.4 mL of liquid insecticide was added to a glass tube with a diameter of 1.9 cm and a length of 7.2 cm. Distilled water in a tube was used as an untreated control. Using a hot-dog roller, the tubes were rolled until the liquid was evenly distributed on the wall of the tubes to form a dried drug film. Twenty wasps (10 males and 10 females) were placed into the tube with the drug film for three replicates. The tube mouth was sealed with a cotton plug. After 1 h, the wasps were transferred into a clean finger-shaped tube and fed 10% honey water. The survival of wasps was observed 24 h later. All the wasps in this experiment were placed in a climate-controlled growth chamber at 25 ± 0.5 °C, 60 ± 0.5% relative humidity, and a 16 h:8 h photoperiod. The dose–mortality relationships and 50% lethal concentrations (LC_50_) of each insecticide for *T. drosophilae* adults were determined using a log-probit model at *p* > 0.05. The safety factor was calculated according to the following formula: safety factor (SF) = (LC_50_)/(Max recommended field dose). Agents were assigned to four risk levels based on the safety factor: (1) sky-high: safety factor ≤ 0.05; (2) high: 0.05 < safety factor ≤ 0.5; (3) medium: 0.5 ≤ safety factor ≤ 5; (4) low: safety factor > 5 [29].

### 2.5. Effects of Insecticides on the Parasitism Rate of Trichopria drosophilae

The aforementioned LC_10_ and LC_50_ values of eight insecticides for *D. suzukii* larvae were used to investigate the toxicity on *T. drosophilae* offspring reared on contaminated hosts. Chemical exposure was performed by allowing *T. drosophilae* females to parasitize 1-day-old *D. suzukii* pupae exposed to insecticides in two ways. (1) Exposure of host larvae: second-instar *D. suzukii* larvae were reared on an artificial diet soaked for 2 min in insecticides at the LC_10_ and LC_50_ concentrations; second-instar *D. suzukii* larvae reared on an untreated artificial diet were the control. (2) Exposure of host pupae: Twenty 1-day-old pupae were soaked for 2 min in each insecticide liquid at the LC_10_ and LC_50_ concentrations. Pupae soaked in distilled water were the untreated control. Air-dried pupae were used for this experiment.

Twenty contaminated *D. suzukii* pupae in both methods were parasitized for 24 h by ten 5-day-old *T. drosophilae* pairs in clear plastic tubes (9.5 cm height and 1.5 cm diameter). A 30% honey solution in tubes was used to feed *T. drosophilae*. All the wasps in this experiment were placed in a climate-controlled growth chamber at 25 ± 0.5 °C, 60 ± 0.5% relative humidity, and a 16 h:8 h photoperiod. Four days later, *D. suzukii* had emerged from the non-parasitized pupae. The remaining intact pupae were collected and observed under a stereomicroscope. The pupae with obvious oviposition holes were regarded as successfully parasitized by *T. drosophilae*, and were used to calculate the parasitism rate of *T. drosophilae*. The eclosion rate of *T. drosophilae* exposed to insecticides was the proportion of emerging offsprings in the 20 total tested pupae in each treatment. Tubes without wasps were used for the control treatment. Three replicates were carried out for each treatment.

### 2.6. Data Analysis

The dose–mortality relationships and the 10% and 50% lethal concentrations (LC_10_ and LC_50_) of each insecticide for larvae and adults of *D. suzukii* and *T. drosophilae* adults were determined using a log-probit model at *p* > 0.05. Raw data were analyzed for normality and homogeneity of variances using the Kolmogorov–Smirnov test.

One-way analysis of variance (ANOVA) 16.0 was used to analyze the survival rates of *D. suzukii* pupae exposed to insecticides at the LC_10_ and LC_50_ and the parasitism rate of *T. drosophilae*. Tukey’s multiple comparison tests were performed, with *p* < 0.05 indicating significance.

## 3. Results

### 3.1. Toxicity of Eight Insecticides on Drosophila suzukii

#### 3.1.1. Toxicity at the Larval Stage

The toxicological data for each insecticide for *D. suzukii* larvae evaluated using a probit analysis are shown in Table 3. Among the eight insecticides, emamectin benzoate was the most toxic for *D. suzukii*, killing 50% and 10% of the tested population at 0.0213 mg/L and 0.0048 mg/L, respectively. The next most toxic insecticides were spinetoram, lambda-cyhalothrin, abamectin, and sophocarpidine, followed by chlorantraniliprole and imidacloprid. Azadirachtin was the least toxic compound with a 50% lethal concentration of 526.55 mg/L and a 10% lethal concentration of 67.34 mg/L. The 50% and 10% lethal concentrations of each insecticide in Table 3 were used for feeding *D. suzukii* larvae to pupae and for estimating the effects of insecticides on the parasitism rate of *T. drosophilae*.

#### 3.1.2. Toxicity at the Pupal Stage

**Figure 1:**
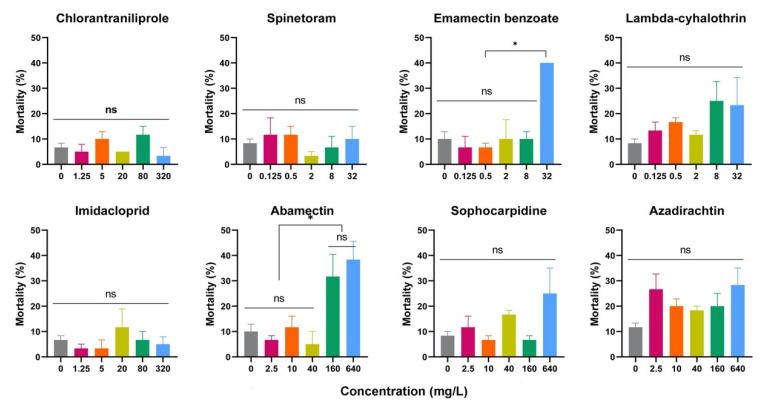
The mortality of *Drosophila suzukii* pupae exposed to eight insecticides with different concentrations. One-way ANOVA followed by Tukey’s multiple comparison test was performed with a significant difference at * *p* < 0.05, ns *p* > 0.05. Error bars represent the standard error of the mean.

The toxicological data for each insecticide for *D. suzukii* pupae are shown in Figure 1. These eight insecticides showed low toxicity on pupae at the present concentrations. Therefore, probit analysis was difficult. The mortality of pupae exposed to chlorantraniliprole (*p* = 0.259), spinetoram (*p* = 0.705), lambda-cyhalothrin (*p* = 0.315), imidacloprid (*p* = 0.667), sophocarpidine (*p* = 0.101), and azadirachtin (*p* = 0.087) at different concentrations did not differ significantly from that of the control group. Though the mortality of pupae exposed to emamectin benzoate at 32 mg/L was significantly higher than that of the control (*p* = 0.001), there were no significant differences in mortality between emamectin benzoate at concentrations from 0.125 mg/L to 8 mg/L and the control group. The mortality of pupae exposed to abamectin at 160 mg/L and 640 mg/L was significantly higher than that of the control group (*p* = 0.004); however, there were no significant differences in mortality between abamectin at concentrations of 2.5 mg/L to 40 mg/L and the control group.

#### 3.1.3. Toxicity at the Adult Stage

The toxicological data for each insecticide for *D. suzukii* adults evaluated in a probit analysis are shown in Table 4. Among the eight insecticides, spinetoram and lambda-cyhalothrin were the most toxic for *D. suzukii*, killing 50% of the tested population at concentrations of 0.32 mg/L and 0.80 mg/L, respectively. Chlorantraniliprole, abamectin, sophocarpidine, and azadirachtin were less toxic for *D. suzukii,* with 50% lethal concentrations of 61.68, 48.58, 67.49, and 56.74, respectively.

### 3.2. Acute Toxicity of Eight Insecticides on Trichopria drosophilae

**Figure 2:**
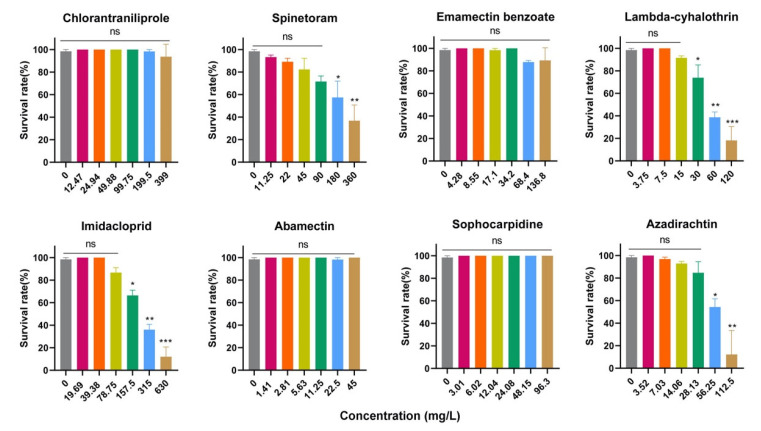
The survival rate of *Trichopria drosophilae* adults exposed to eight insecticides with different concentrations. One-way ANOVA followed by Tukey’s multiple comparison test was performed with a significant difference at *** *p* < 0.0001, ** *p* < 0.001, * *p* < 0.05, ns *p* > 0.05. Error bars represent the standard error of the mean.

The acute toxicity of studied insecticides on *T. drosophilae* is shown in Figure 2. Compared with those of the control, the survival rates of *T. drosophilae* adults exposed to chlorantraniliprole (*p* = 0.566), emamectin benzoate (*p* = 0.136), abamectin (*p* = 0.563), and sophocarpidine (*p* = 0.463) did not differ significantly. Six insecticides had low acute toxicities to *T. drosophilae* adults. High toxicity to *T. drosophilae* adults was found for high concentrations of spinetoram (*p* < 0.001), lambda-cyhalothrin (*p* < 0.001), imidacloprid (*p* < 0.001), and azadirachtin (*p* < 0.001). The mortality of *T. drosophilae* adults after 1 h of treatment with field concentrations of spinetoram, lambda-cyhalothrin, and imidacloprid were approximately 15%, 9%, and 40%, respectively. According to the log-probit analysis at the *p* > 0.05 level of dose–mortality relationships, the 50% lethal concentrations (LC_50_) of the four most toxic insecticides for *T. drosophilae* adults are shown in Table 5. The safety factors of lambda-cyhalothrin and imidacloprid were 4.4 and 1.4, respectively, indicating a medium risk level for *T. drosophilae* adults. Spinetoram and azadirachtin were classified as low risk with safety factors of 6.5 and 5.0.

### 3.3. Effects of Insecticides on Parasitism and Eclosion Rates of Trichopria drosophilae

**Figure 3:**
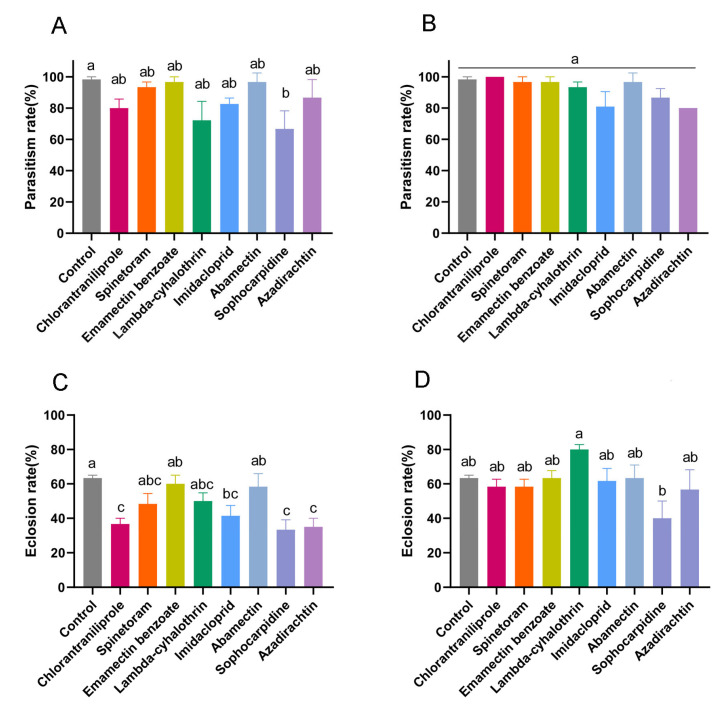
The effects of eight insecticides on parasitism rate ((**A**) LC_50_; (**B**) LC_10_) and eclosion rate ((**C**) LC_50_; (**D**) LC_10_) of *Trichopria drosophilae* determined by treating *Drosophila suzukii* larvae with insecticides at LC_50_ and LC_10_ concentrations. One-way ANOVA followed by Tukey’s multiple comparison test was performed with a significant difference at *p* < 0.05. Error bars represent the standard error of the mean. Different letters on the bars indicate significant differences among treatments.

LC_10_ and LC_50_ values of eight insecticides previously determined for *D. suzukii* larvae (Table 3) were used to estimate the effects on parasitism and eclosion rates of *T. drosophilae*. Two methods were used for this experiment. First, host larvae treated with eight insecticides at the LC_50_ and LC_10_ concentrations were reared to the pupal stage (Figure 3). Compared with that of the control (98.3 ± 1.7%), the parasitism rate of *T. drosophilae* was significantly lower after treatment with sophocarpidine at the LC_50_ concentration (66.7 ± 6.7%; *p* = 0.010). However, there were no significant differences in parasitism rates for the other seven insecticides (Figure 3A). The eclosion rates of *T. drosophilae* exposed to chlorantraniliprole, imidacloprid, sophocarpidine, and azadirachtin at the LC_50_ concentration were reduced to 36.7 ± 3.3%, 41.5 ± 6.0%, 33.3 ± 3.3%, and 35.0 ± 2.9%, respectively, compared with that of the control (63.3 ± 1.7%; *p* < 0.001) (Figure 3C). The parasitism rate of *T. drosophilae* was not affected by exposure to eight insecticides at the LC_10_ concentration (*p* = 0.053) (Figure 3B). Only sophocarpidine at the LC_10_ concentration reduced the eclosion rate of *T. drosophilae* (40.0 ± 5.8%) significantly (*p* = 0.005) (Figure 3D).

**Figure 4:**
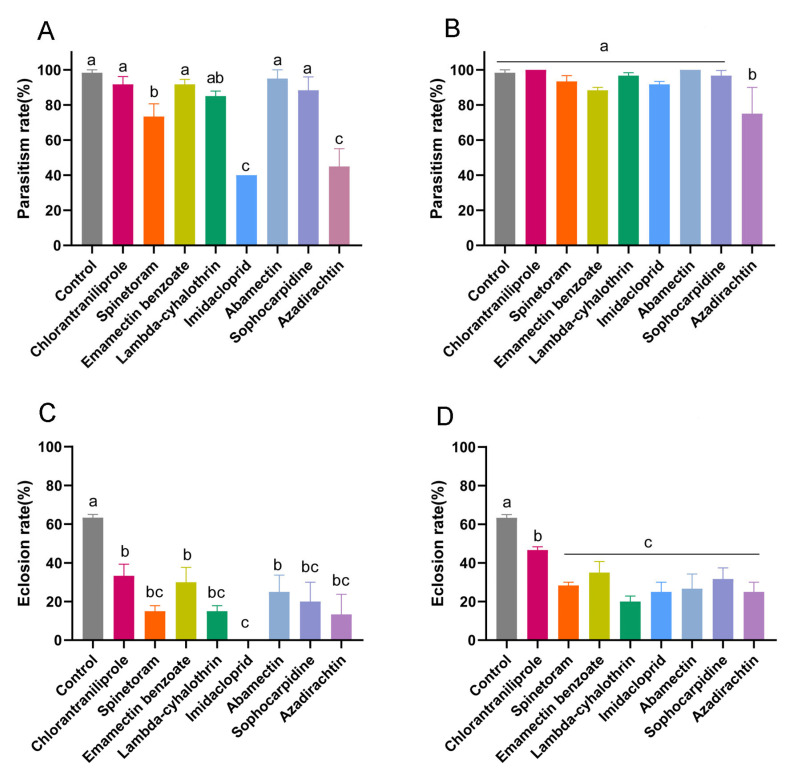
The effects of eight insecticides on parasitism rate ((**A**) LC_50_; (**B**) LC_10_) and eclosion rate ((**C**) LC_50_; (**D**) LC_10_) of *Trichopria drosophilae* determined by treating *Drosophila suzukii* pupae indirectly with insecticides at LC_50_ and LC_10_ concentrations. One-way ANOVA followed by Tukey’s multiple comparison test was performed with a significant difference at *p* < 0.05. Error bars represent the standard error of the mean. Different letters on the bars indicate significant differences among treatments.

Second, the pupae were treated directly with eight insecticides at the LC_10_ and LC_50_ concentrations. The parasitism rates and eclosion rates of *T. drosophilae* were lower than those for the treated larvae (Figure 4). Compared with those of the control, parasitism rates of *T. drosophilae* exposed to spinetoram, imidacloprid, and azadirachtin at the LC_50_ concentration were reduced to 73.3 ± 7.3%, 40.0 ± 0.0%, and 45.0 ± 5.8%, respectively (Figure 4A). Only azadirachtin at the LC_10_ concentration reduced the parasitism rate of *T. drosophilae* (75.0 ± 8.7%) significantly (*p* = 0.001) (Figure 4B). All eight insecticides at the LC_50_ and LC_10_ concentrations reduced the eclosion rate significantly (LC_50_: *p* < 0.001; LC_10_: *p* < 0.001) (Figure 4C,D). Imidacloprid at the LC_50_ concentration applied to pupae led to no wasps emerging (Figure 4C). Therefore, treating *D. suzukii* pupae directly had greater effects on the parasitism and eclosion rates of *T. drosophilae* than treating *D. suzukii* larvae.

## 4. Discussion

The eight insecticides evaluated in this study had higher toxicities to *D. suzukii* larvae than adults according to the 50% lethal concentration. These results were consistent with those of previous studies; for example, the susceptibilities of *D. suzukii* larvae to emamectin benzoate, chlorantraniliprole, and lambda-cyhalothrin were higher than those of adults [12]. Spirotetramat reduced the survival rate of *D. suzukii* adults but was lethal to larvae [13]. Lambda-cyhalothrin, spinosad, and spinetoram showed inhibitory effects on the development of *D. suzukii* larvae [26]. Therefore, the response of insects to insecticides was related to the insect stage, but the field validation for these insecticides is necessary in future studies, as it is beneficial for choosing the appropriate control period of pests during the field application of insecticides [30].

Among the eight insecticides in our study, emamectin benzoate, spinetoram, lambda-cyhalothrin, abamectin, and sophocarpidine had high toxicity to *D. suzukii* larvae. Adults were more sensitive to emamectin benzoate, spinetoram, and lambda-cyhalothrin than to the other five insecticides. Therefore, three insecticides (i.e., emamectin benzoate, spinetoram, and lambda-cyhalothrin) could be used as effective agents for controlling *D. suzukii*. As a spinosyn insecticide, spinetoram has highly effective insecticidal activity against various lepidopterous and dipterous pests and low toxicity against non-target insects [31]. Lambda-cyhalothrin is one of the most common pyrethroids used for agricultural and household pest control, acting through contact and the nervous system [32]. Spinetoram and lambda-cyhalothrin are widely used for controlling *D. suzukii* [7,11,16,33]. Moreover, as a new type of highly efficient antibiotic insecticide based on abamectin B_1_, emamectin benzoate is widely used for pest control [34]. However, it is rarely used for controlling *D. suzukii,* except for several reports in China [12,35]. Whether insecticides can be promoted in the field depends on the toxicity against target pests and the safety of natural enemies. Therefore, further studies are needed to determine whether emamectin benzoate can be used for controlling *D. suzukii* in the field, and to evaluate its safety against parasitic wasps.

An important method for evaluating the safety of insecticides against parasitic wasps is the acute toxicity test, in which wasps are exposed to insecticides indirectly for a short time (GB/T 31270.12-2014, Part 17). The acute toxicity test has been commonly used to evaluate the toxicity of pesticides to *Trichogramma* spp. (Hymenoptera: Trichogrammatidae) in China [29,36,37]. *T. drosophilae* is an important pupal parasitoid of *Drosophila melanogaster* (Diptera: Drosophilidae) and *D. suzukii*, laying a single egg per oviposition inside pupae [17]. In this study, based on the median lethal rate (LC_50_) and safety factor (SF), lambda-cyhalothrin and imidacloprid were identified as medium risk and the other six insecticides were identified as low risk against *T. drosophilae* adults. In particular, the mortality rate of *T. drosophilae* adults after 1 h of treatment with field concentrations of imidacloprid was about 40%. Although the effect of lambda-cyhalothrin and imidacloprid on *T. drosophilae* in the field needs to be verified in future studies, these two insecticides should also be used with caution in integrated pest management programs against *D. suzukii*.

In addition to the increased mortality caused by indirect contact with insecticides, the long-term, trans-generational effects of sublethal doses of pesticides on parasitic wasps should also be considered when assessing the safety of pesticides [23,26]. Treating host larvae or pupae with insecticides can affect the parasitic behavior of parasitoids and the emergence of the next generation. For example, spinosad applied to host insects at the late larval and pupal stages reduced adult emergence for two species of *Trichogramma* significantly [38]. Lambda-cyhalothrin applied at the pre-pupal and pupal stages and spinosad applied to pre-pupae significantly reduced the adult emergence of *Trichogramma galloi* (Hymenoptera: Trichogrammatidae) [23]. For two pupal parasitoids of *D. suzukii*, the sensitivity to dry residues of eight commercial insecticides was higher for *P. vindemmiae* than for *T. anastrephae*, resulting in a significant reduction in parasitism for the former species [21]. Sublethal doses of cyazypyr and dimethoate negatively affected the success of parasitism and the number of progeny of *T. drosophilae* [26]. The results of our study were consistent with those of these previous reports. In our study, although the insecticides showed no toxicity against *D. suzukii* pupae at the sublethal dose (LC_10_) and LC_50_, the direct exposure of larvae and pupae to some insecticides could decrease the parasitism and eclosion rates of *T. drosophilae*. When *D. suzukii* larvae were exposed to sophocarpidine at the LC_50_ concentration, the parasitism rate of *T. drosophilae* adults decreased by 32.21%. The eclosion rates of *T. drosophilae* in the next generation were reduced by chlorantraniliprole, imidacloprid, sophocarpidine, and azadirachtin at the LC_50_ concentration and sophocarpidine at the sublethal dose. When *D. suzukii* pupae were exposed to insecticides, the parasitism rates of *T. drosophilae* adults were reduced by spinetoram, imidacloprid, and azadirachtin at the LC_50_ concentration. The sublethal doses of all of the insecticides decreased the eclosion rate of the next generation but did not affect the parasitism rates of *T. drosophilae* adults. These results also suggested that the semilethal and sublethal doses of insecticides had greater effects on the eclosion rate of *T. drosophilae* than on the parasitism rates of the F_0_ generation. Eggs successfully laid in *D. suzukii* pupae might not develop into adults.

Screening highly effective insecticides is critical to improve integrated pest management strategies for *D. suzukii* through monitoring the susceptibility and resistance of *D. suzukii* to insecticides. Mertz et al. [39] examined the toxicity of 19 alternative insecticides to a susceptible *D. melanogaster* strain and cross-resistance using a field-collected population. There were high levels of resistance to zeta-cypermethrin, malathion, and acetamiprid in all populations sampled over 33 months. Moreover, Gress et al. [40] presented a simple, cost-effective tool for assaying the resistance of *D. suzukii* in commercial caneberry fields to three commonly used insecticides (malathion, spinosad, and zeta-cypermethrin). A more convenient and effective assaying method is conducive to the study of resistance monitoring. Therefore, monitoring the resistance of *D. suzukii* to insecticides and selecting resistant insect lines is important for resistance management research and will be a focus of our future study.

## 5. Conclusions

In summary, the eight insecticides had higher toxicities to *D. suzukii* larvae with lower LC_50_ values than those for adults. Lambda-cyhalothrin and imidacloprid caused mortality in *T. drosophilae* adults. For some insecticides that were not lethal to *T. drosophilae* adults at the recommended dose in the field (e.g., chlorantraniliprole, emamectin benzoate, sophocarpidine, abamectin, azadirachtin, and spinetoram), the semilethal and sublethal doses may affect the parasitism rate of the F_0_ generation and the eclosion rate of the next generation. The toxicity of insecticides against *D. suzukii* and *T. drosophilae* in the field needs to be verified in future studies. The results of this study improve our understanding of the effects of insecticide residues on *T. drosophilae* populations and provide a basis for the development of scientific and efficient measures to manage *D. suzukii* and protect *T. drosophilae*.

## Figures and Tables

**Table 1:** Information on test insecticides used in this study.

Insecticide Chemical Class	Active Ingredient	Concentration of Active Ingredient (g/L)	Production Enterprise	Target Pest	Crop	Active Ingredient Max Recommended Field Dose (g/ha)
Diamides	Chlorantraniliprole	350	FMC Corporation Shanghai Agricultural Technology Co., Ltd. Shanghai, China	Fruit moths	Peach and apple	39.90
Spinosyns	Spinetoram	60	Codihua Agricultural Technology Co., Ltd. Weifang, China	Fruit flies	Waxberry	36.00
Avermectins	Emamectin benzoate	50	Shandong Jingbo Agrochemical Technology Co., Ltd. Shanghai, China	Trips	Mango	13.68
Pyrethroids	Lambda-cyhalothrin	25	Syngenta (Nantong) Crop Protection Co., Ltd. Nantong, Chian	Fruit moths and aphids	Orange and apple	12.00
Neonicotinoids	Imidacloprid	700	Bayer Crop Science (China) Co., Ltd. Hangzhou, China	Fruit moths and aphids	Apple	63.00
Avermectins	Abamectin	180	Zhejiang Shijia Technology Co., Ltd. Hangzhou, China	Fruit moths and spider mites	Orange and apple	4.50
Botanical insecticides	Sophocarpidine	15	Chengdu New Chaoyang Crop Science Co., Ltd. Chengdu, China	Aphids	Grape, orange, and strawberry	9.63
Botanical insecticides	Azadirachtin	3	Chengdu LvJin Biotechnology Co., Ltd. Chengdu, China	Aphids	Vegetables and strawberry	11.25

**Table 2:** The concentrations of eight insecticides applied in the toxicity experiments.

Active Ingredient	Concentration (mg/L)			
	*D. suzukii* Larvae	*D. suzukii* Pupae	*D. suzukii* Adults	*Trichopria drosophilae*
Chlorantraniliprole	160, 120, 80, 40, 20, 10	320, 80, 20, 5, 1.25	640, 320, 160, 80, 40, 20	399, 199.5, 99.75, 49.88, 24.94, 12.47
Spinetoram	2, 1, 0.5, 0.25, 0.125, 0.0625	32, 8, 2, 0.5, 0.125	2, 1, 0.5, 0.25, 0.125, 0.0625	360, 180, 90, 45, 22, 11.25
Emamectin benzoate	0.1, 0.05, 0.025, 0.0125, 0.0625, 0.03125	32, 8, 2, 0.5, 0.125	16, 8, 4, 2, 1, 0.5	136.8, 68.4, 34.2, 17.1, 8.55, 4.28
Lambda-cyhalothrin	4, 2, 1, 0.5, 0.25, 0.125	32, 8, 2, 0.5, 0.125	8, 4, 2, 1, 0.5, 0.25	120, 60, 30, 15, 7.5, 3.75
Imidacloprid	320, 160, 80, 40, 20, 10	320, 80, 20, 5, 1.25	80, 40, 20, 10, 5, 2.5	630, 315, 157.5, 78.75, 39.38, 19.69
Abamectin	2, 1, 0.5, 0.25, 0.125, 0.0625	640, 160, 40, 10, 2.5	160, 120, 80, 40, 20, 10	45, 22.5, 11.25, 5.63, 2.81, 1.41
Sophocarpidine	3.2, 1.6, 0.8, 0.4, 0.2, 0.1	640, 160, 40, 10, 2.5	320, 160, 80, 40, 20, 10	96.3, 48.15, 24.08, 12.04, 6.02, 3.01
Azadirachtin	1200, 960, 640, 160, 40, 10	640, 160, 40, 10, 2.5	320, 160, 80, 40, 20, 10	112.5, 56.25, 28.13, 14.06, 7.03, 3.52

**Table 3:** Probit analysis for the toxicity of eight insecticides on *Drosophila suzukii* larvae.

Active Ingredient	Probit Model	SE (Slope)	SE (Intercept)	LC_50_ (95% Confidence Interval)	LC_10_ (95% Confidence Interval)	R^2^	Chi-Square	*p* Value
Chlorantraniliprole	Y = 2.01X − 3.03	0.20	0.34	32.09 (19.46–46.78)	7.41 (1.92–13.62)	0.98	9.16	0.57
Spinetoram	Y = 1.39X + 0.90	0.16	0.11	0.23 (0.17–0.29)	0.027 (0.013–0.045)	0.93	4.82	0.31
Emamectin benzoate	Y = 1.99X + 3.32	0.20	0.34	0.021 (0.014–0.032)	0.0048 (0.0017–0.0081)	0.92	8.14	0.09
Lambda-cyhalothrin	Y = 1.54X + 0.50	0.16	0.084	0.47 (0.37–0.59)	0.07 (0.037–0.11)	0.99	0.91	0.92
Imidacloprid	Y = 0.96X − 1.52	0.14	0.26	38.31 (25.82–53.38)	1.79 (0.42–4.057)	0.99	0.45	0.98
Abamectin	Y = 2.30X + 1.05	0.24	0.13	0.35 (0.29–0.42)	0.10 (0.063–0.13)	0.98	6.00	0.20
Sophocarpidine	Y = 2.40X + 0.80	0.23	0.11	0.46 (0.39–0.55)	0.14 (0.094–0.18)	0.98	0.95	0.57
Azadirachtin	Y = 1.43X − 3.90	0.15	0.41	526.55 (305.02–1000.44)	67.34 (13.85–138.29)	0.95	8.72	0.07

**Table 4:** Probit analysis for the toxicity of eight insecticides on *Drosophila suzukii* adults.

Active Ingredient	Probit Model	SE (Slope)	SE (Intercept)	LC_50_	95% Confidence Interval	R^2^	Chi-Square	*p* Value
Chlorantraniliprole	Y = 1.52X − 2.72	0.14	0.28	61.68	38.62–90.14	0.99	7.41	0.12
Spinetoram	Y = 2.76X + 1.37	0.23	0.14	0.32	0.28–0.37	0.99	3.58	0.47
Emamectin benzoate	Y = 2.03X − 0.74	0.20	0.13	2.31	1.88–2.75	0.99	2.13	0.71
Lambda-cyhalothrin	Y = 2.97X + 0.29	0.25	0.084	0.80	0.71–0.90	0.98	4.64	0.33
Imidacloprid	Y = 3.99X − 3.62	0.37	0.36	8.11	7.17–9.13	0.99	1.19	0.88
Abamectin	Y = 2.31X − 3.90	0.19	0.33	48.58	35.22–65.60	0.95	8.71	0.07
Sophocarpidine	Y = 1.77X − 3.23	0.16	0.31	67.49	55.46–82.37	0.96	4.85	0.30
Azadirachtin	Y = 2.38X − 4.17	0.19	0.34	56.74	48.99–65.81	0.98	6.29	0.18

**Table 5:** Acute toxicity of four insecticides on *Trichopria drosophilae* adults.

Active Ingredient	Probit Model	SE (Slope)	SE (Intercept)	LC_50_	95% Confidence Interval	R^2^	Safety Factor	Risk Level	Chi-Square	*p* Value
Spinetoram	Y = 1.34X − 3.25	0.19	0.40	266.98	195.60–415.12	0.99	6.5	Low	0.52	0.97
Lambda-cyhalothrin	Y = 2.81X − 5.01	0.29	0.52	60.41	51.92–71.10	0.99	4.4	Medium	3.39	0.47
Imidacloprid	Y = 1.40X − 2.80	0.13	0.28	100.58	75.23–135.91	0.99	1.4	Medium	3.59	0.46
Azadirachtin	Y = 3.30X − 5.96	0.45	0.82	63.99	54.94–74.68	0.97	5.0	Low	4.24	0.37

Risk level: sky-high, safety factor < 0.05. Risk level: high, 0.05 < safety factor < 0.5. Risk level: medium, 0.05 < safety factor < 5. Risk level: low, safety factor > 5.

## Data Availability

The raw data generated during the experiment will be made available to interested parties with a reasonable timeline for the request.

## Supplementary Materials

The following supporting information can be downloaded at https://www.mdpi.com/article/10.3390/insects15110910/s1: Table S1: The raw data of survival rate of D. suzukii larvae exposed to eight insecticides; Table S2: The raw data of survival rate of *D. suzukii* adults exposed to eight insecticides.
